# The exact synchronization timing between the cleavage embryo stage and duration of progesterone therapy-improved pregnancy rates in frozen embryo transfer cycles: A cross-sectional study

**DOI:** 10.18502/ijrm.v19i3.8570

**Published:** 2021-03-21

**Authors:** Marjan Omidi, Iman Halvaei, Fatemeh Akyash, Mohammad Ali Khalili, Azam Agha-Rahimi, Leila Heydari

**Affiliations:** ^1^Research and Clinical Center for Infertility, Yazd Reproductive Sciences Institute, Shahid Sadoughi University of Medical Sciences, Yazd, Iran.; ^2^Department of Anatomical Sciences, Faculty of Medical Sciences, Tarbiat Modares University, Tehran, Iran.; ^3^Stem Cell Biology Research Center, Yazd Reproductive Sciences Institute, Shahid Sadoughi University of Medical Science, Yazd, Iran.

**Keywords:** Endometrium, Embryo transfer, Pregnancy, Live birth, Progesterone.

## Abstract

**Background:**

Synchronization between the embryonic stage and the uterine endometrial lining is important in the outcomes of the vitrified-warmed embryo transfer (ET) cycles.

**Objective:**

The aim was to investigate the effect of the exact synchronization between the cleavage stage of embryos and the duration of progesterone administration on the improvement of clinical outcomes in frozen embryo transfer (FET) cycles.

**Materials and Methods:**

312 FET cycles were categorized into two groups: (A) day-3 ET after three days of progesterone administration (n = 177) and (B) day-2 or -4 ET after three days of progesterone administration (n = 135). Group B was further divided into two subgroups: B1: day-2 ET cycles, that the stage of embryos were less than the administrated progesterone and B2: day-4 ET cycles, that the stage of embryos were more than the administrated progesterone. The clinical outcome measures were compared between the groups.

**Results:**

The pregnancy outcomes between groups A and B showed a significant differences in the chemical (40.1% vs 27.4%; p = 0.010) and clinical pregnancies (32.8% vs 22.2%; p = 0.040), respectively. The rate of miscarriage tended to be higher and live birth rate tended to be lower in group B than in group A. Also, significantly higher rates were noted in chemical pregnancy, clinical pregnancy, and live birth in group A when compared with subgroup B2.

**Conclusion:**

Higher rates of pregnancy and live birth were achieved in day-3 ET after three days of progesterone administration in FET cycles.

## 1. Introduction 

For implantation and pregnancy establishment, a normal developing embryo interacts successfully with a receptive endometrium (1). During endometrial receptivity, the window of implantation (WOI) is opened and temporally complex changes, including the morphological, cytoskeletal, biochemical, and genetic, take place in the endometrium (2). WOI is open five days after exposure to endogenous or exogenous progesterone and restricted only for two days. When the embryo reaches the blastocyst stage and is ready for implantation, the endometrium should be in WOI phase. Therefore, if there is no synchronization, the embryo implantation does not occur (3). The “synchrony” is defined as when the embryo and the uterus are both developing at the same rate; so, they will be ready to cross-talk for a successful implantation (4). One of the main cause of recurrent implantation failure is displacement of WOI that causes an asynchronous between embryo and endometrium (5).

Cryopreservation of human embryos and subsequent frozen embryo transfer (FET) has become the routine practice in modern ART. Previously, the pregnancy rates following FET cycles were lower compared to the fresh embryo transfer (ET) cycles, due to the suboptimal embryo survival after slow freezing (6, 7). The cryopreservation methods were improved by introduction of vitrification, which increased the cryo-survival rates up to 95% (8, 9). The degree of embryo development and endometrial synchronization can influence the chance of embryo implantation in ART program (10). Although, the pregnancy rate was enhanced by improving the embryo quality during FET cycles (11), little is known about the optimization of the synchronization between the endometrial receptivity and embryo developmental stage.

Several randomized trials reported the effect of the different endometrial preparation methods on the outcomes of FET cycles (1, 12, 13). The common method of endometrial preparation for FET cycles is administration of estrogen until the endometrial thickness of about 0.8-1.4 cm, then adding progesterone for the number of days proportional to the stage of the embryos being transferred (10). The duration of progesterone therapy is critical for establishing the WOI. However, this optimal duration of progesterone administration before FET has not been established (14). There is an assumption that after estrogen priming, the number of days for exposure to progesterone will affect an appropriate endometrial lining to support the implantation of the blastocyst. However, endometrial biopsy in natural cycles showed over two-days differences between the histologic dating and the actual day after ovulation in 5-50% of the patients (15, 16).

Therefore, the aim of this study was to investigate the effect of ET in different stages of embryo development after three days of progesterone administration on the clinical outcomes in FET cycles. To the best of our knowledge, this is the first preliminary investigation comparing the clinical outcomes of FET in different stages of embryo development after three days of progesterone administration.

## 2. Materials and Methods 

### Study design 

This study was conducted at the Yazd Reproductive Sciences Institute, Yazd, Iran. In this study, patients qualified for elective FET. Cycles were evaluated between January 2018 and August 2019. A total of 312 elective FET cycles met the inclusion criteria for this study. The cycles were included if high-quality embryos (grades A and B) were transferred. Egg donation, surrogacy, in vitro maturation, and blastocyst transfer cycles were excluded. Cycles were also excluded if the patients were lost to follow-up regarding the eventual occurrence of the pregnancy or delivery. The cycles were categorized based on the synchronization of the prepared endometrium and transferred embryo(s) regarding the duration of progesterone administration and the stage of the cleavage embryos. So, the cycles were divided into two groups: (A) day-3 ET after three days of progesterone administration (n = 177) and (B) day-2 or -4 ET after three days of progesterone administration (n = 135). While analyzing, group B was further divided into two subgroups: B1: day-2 embryo after three days of progesterone therapy and B2: day-4 embryo after three days of progesterone therapy. In all groups, the endometria were exposed to progesterone for three days.

### Ovarian stimulation 

GnRH antagonist protocol was conducted in women undergoing stimulation cycles for IVF or ICSI. Ovarian stimulation with Gonal-f 150 IU (SA Merck Serono, Geneva, Switzerland) was initiated on day 2 of the cycle subcutaneously and later 0.25 mg of cetrorelix (Cetrotide; Asta Medica, Frankfurt, Germany) daily was added when the leading follicle reached 14 mm in diameter. Ovulation was routinely triggered with 10,000 IU HCG. Oocyte retrieval was performed 34-36 hr later (17).

### Endometrium preparation

The artificial cycles were carried out using 6 mg daily oral estradiol valerate (2 mg, Aburaihan Co., Tehran, Iran) from the second day of the menstrual cycle. On days 12-13 of the cycle, ultrasonography was done. The vaginal progesterone (Cyclogest; Actavis, UK limited, England) was given 400 mg twice daily and oral estradiol was continued when the endometrial thickness was ≥ 8 mm. ET was performed after three days of progesterone exposure, and on the day of ET, the fourth vaginal progesterone was administered. Estradiol valerate and progesterone supplementations were continued for two weeks after ET, and if the serum βHCG was positive, the hormone supplementations were continued until twelve 12 wk of gestation.

### Embryo vitrification 

The vitrification was done according to Vitrolife kit (Vitrolife Inc., Gothenburg, Sweden). Briefly, the embryos were equilibrated into the Vitri 1${}^{\mathrm{TM}}$ Cleave solution for 5 min, and in Vitri2${}^{\mathrm{TM}}$ Cleave solution for 2 min. finally the embryo were placed into Vitri 3${}^{\mathrm{TM}}$ Cleave solution for 30 sec and loaded on the top of cryotop. The cryotop was submerged directly to liquid nitrogen and store it. For warming, the top of cryotop was submerged to Warm1${}^{\mathrm{TM}}$ solution and then placed in Warm2 and 3${}^{\mathrm{TM}}$. Finally washed in Warm4${}^{\mathrm{TM}}$ solution and culture in G1 medium (Vitrolife Inc., Gothenburg, Sweden).

### Evaluation of embryo morphology

The morphology of the embryos was assessed at the same time of ET as described elsewhere (18). The grade A embryos were with regular size blastomere, and no fragmentation; grade B, the embryos withuneven blastomeres and/or less than 10% fragmentation; grade C, the embryos with more than 10% fragmentation but no more than 25% of blastomeres. Embryos with grades A and B were considered as top quality embryos

A positive beta hCG test (30-100 mIU/mL; Monobind Inc., CA, USA) after fourteenth of ET day was considered positive chemical pregnancy. If a fetal heart beat was observed by ultrasonography at the end of the seventh week of gestation the clinical pregnancy was considered positive. The chemical and clinical pregnancy rate was calculated by dividing the total number of the pregnancies by the number of transfer. The total number of clinical pregnancy losses before the 20${}^{\mathrm{th}}$ wk of gestation divided by the total number of clinical pregnancies was defined as the miscarriage rate. The live birth rate was calculated the total number of deliver per number of transfers.

### Ethical considerations

This retrospective cohort study was approved by the ethics committee of Yazd Reproductive Sciences Institute (code: IR.SSU.RSI.REC.1396.10). Patients' data were collected confidentially.

### Statistical analysis

Data were analyzed using the statistical package for the Social Sciences (SPSS, Version 14, IBM, USA). Quantitative and qualitative data are presented as Mean ± SD and percentages, respectively. Mann-Whitney U-test, independebt *t* test and the Chi-square test were applied to the quantitative and qualitative data, respectively. All tests were two-tailed, and p < 0.05 considered as significant.

## 3. Results

A total of 611 high-quality embryos from 312 FET cycles were transferred. No significant differences were found in female age and the cause of infertility between the groups (Tables I, II). Also, insignificant differences were seen regarding the number of transferred embryos between groups A and B (p = 0.800, Table I). In all cycles, there was no differences in the number of high-quality transferred embryos between the groups (p = 0.850). Data also showed that the rates of chemical and clinical pregnancies between two groups were significantly different (p = 0.020 and p = 0.040, respectively) (Table I). In addition, the rates of miscarriage tended to be higher and delivery rates tended to be lower in group B (16.6% vs 15.5%, p = 0.800) than in group A (18.5% vs 27.6%, p = 0.100), respectively.

For further analysis, the laboratory and clinical characteristics of FET cycles were compared between the three groups categorized according to synchronization of the embryo stage and prepared endometrium (Table II). The female age, cause of infertility, number of embryos transferred, and embryo quality were matched between the three groups. Furthermore, the chemical pregnancy, clinical pregnancy, and live birth rates were significantly lower in group B2 compared to group A (p = 0.003, p = 0.010, and p = 0.020, respectively).

**Table 1:** The comparison of laboratory and clinical data between the two groups


**Variables**		**Group A (n = 177)**	**Group B** **(n = 135)**	**P-value**	**Odds ratio (95% CI)**
Female age (yr)*		29.61 ± 3.9	30.15 ± 4.0	0.200${}^{a}$	
Cause of infertility**			0.200${}^{b}$	
	**Male factor**	44.1	42.2	
	**Female factor**	44.1	38.5	
	**Male and female**	9.5	13.4	
	**Unexplained**	2.3	5.9	
No. of transferred embryos*		1.96 ± 0.3	1.96 ± 0.2	0.800${}^{c}$	
Embryo grade**			
	**A**	19.8	20	
	**B**	80.2	80	0.850${}^{b}$	0.93 (0.46–1.88)
Chemical pregnancy**		71/177 (40.1)	37/135 (27.4)			0.020${}^{b}$	1.77 (1.09–2.87)
Clinical pregnancy**		58/177 (32.8)	30/135 (22.2)	0.0400${}^{b}$	1.7 (1.02–2.85)
Miscarriage rate**		9/58 (15.5)	5/30 (16.6)	0.800${}^{b}$	0.91 (0.27–3.03)
Live birth rate**		49/177 (27.6)	25/135 (18.5)	0.100${}^{b}$	1.68 (97–290)
*Data presented as Mean ± SD. **Data presented as n (%). ${}^{a}$Mann-Whitney test, ${}^{b}$Chi-square test, ${}^{c}$ *t* test and U-tests. (A) Day-3 embryo transfer (ET) after three days of progesterone administration. (B) Day-2 or -4 ET after three days of progesterone administration					

**Table 2:** The comparison of laboratory and clinical data between the three groups


**Variables**		**Group B1 (n = 96)**	**Group B2 (n = 39)**	**Group A (n = 177)**	**P-value**
Female age (yr)${}^{*a}$		30.1 ± 4	30.26 ± 4	29.61 ± 3.9	
	31 (20–36)	30 (23–37)	30 (20–37)	0.400
Cause of infertility${}^{**b}$					
	**Male factor**	45.8	33.4	44.1	
	**Female factor**	36.5	43.6	44.1	
	**Male and female factor **	11.5	17.9	9.5	
	**Unexplained**	6.2	5.1	2.3	0.300
No. of transferred embryos${}^{*c}$		1.93 ± 0.3	2.03 ± 0.1	1.96 ± 0.3		0.200
Embryo grade${}^{**b}$					
	**A**	20.8	17.9	19.8		
	**B**	79.2	82.1	80.2	0.900
Chemical pregnancy${}^{**b}$		29/96 (30.2)	8/39 (20.5)	71/177 (40.1)		0.100${}^{@,\#}$ 0.003${}^{\$}$
Clinical pregnancy${}^{**b}$		23/96 (24)	7/39 (17.9)	58/177 (32.8)	0.100${}^{@}$ 0.200${}^{\#}$ 0.010${}^{\$}$
Miscarriage rate${}^{**b}$		4/23 (17.3)	1/7 (14.2)	9/58 (15.5)	0.800${}^{@}$ 0.500${}^{\#}$ 0.700${}^{\$}$
Live birth rate${}^{**b}$		19/96 (19.7)	6/39 (15.3)	49/177 (27.6)	0.100${}^{@}$ 0.300${}^{\#}$ 0.020${}^{\$}$
*Data presented as Mean ± SD. **Data presented as n (%). ${}^{a}$Mann–Whitney test, ${}^{b}$Chi-square test, ${}^{c}$ *t* test and U-tests (A) Day-3 embryo transfer (ET) after three days of progesterone administration. (B1) Day-2 embryo ET after three days of progesterone therapy. (B2) Day-4 embryo ET after three days of progesterone therapy ${}^{@}$Difference between B1 and A cycles, ${}^{\#}$Difference between B1 and B2 transfer cycles, ${}^{\$}$Difference between B2 and A cycles					

## 4. Discussion

The optimal method for endometrial preparation in FET cycles still remains unclear. Some studies reported natural cycles had better outcomes following the transfer of day-3 frozen embryos (19, 20). However, Wright and colleagues concluded that artificial cycles and stimulated cycles had comparable results about the rates of pregnancy and live birth (21). Recently, Dong and associates stated that utilizing monitoring ovulation regimen is the best method for the determining the day of ET in natural cycles. They reported that in these cycles, determining the precise FET timing by monitoring serum progesterone level after ovulation is an unsuitable method (22). Also, in method of hormone preparations, the length of administration of progesterone was reportedly different (23). Routinely, in artificial method for endometrial preparation, the progesterone supplementation is initiated three days before ET with the chance of pregnancy up to 40.5% (24, 25). Our findings also showed a 40.1% pregnancy rate in cycles in which day-3 embryos were transferred to endometrium exposed to progesterone for three days.

According to our search, this is the first study comparing the clinical outcomes of ET in different stages of development to three-days endometrial preparation in artificial FET cycles. Our results demonstrated a significantly higher rates of chemical and clinical pregnancies in group A with respect to the developmental stage of embryos with the developmental maturation of endometrium. However, differences in the miscarriage and live birth rates between the two groups were not significant with tendency to group A. Our data showed that the rates of live birth were approximately 10% higher in group A when compared to group B. Contrary to our results, Vijver and colleagues demonstrated no significant differences in the pregnancy and implantation rates when day-4 embryos were transferred after three or five days of progesterone administration (26). In their study, the stage of embryo was similar, but in our study the duration of progesterone administration was similar in patients and stage of embryo was different between the groups.

While sub-analyzing, our results showed the higher pregnancy and live birth rates when the embryos were transferred B1 (day-2 ET) rather than B2 (day-4 ET). Similar to our data, Van de Vijver and colleagues found the opposite results, when the duration of progesterone administration was shorter than the embryo stage. They demonstrated a higher early pregnancy loss in FET cycles when day-4 embryos were transferred on the third day after the initiation of progesterone supplementation (26). Sharma and colleagues compared the clinical outcome between the three- and four days progesterone supplementation in day-2 cleavage embryo. Their study showed progesterone supplementation for three days correlated with higher pregnancy and implantation rate (27). Also, Ding and colleagues showed that the implantation rates of frozen-thawed blastocysts on day 6 of progesterone administration was better than day 7 (28). Despite our study, matching between the stage of embryo and endometrium was not evaluated in the aforementioned reports.

Prapas and colleagues showed the highest pregnancy rates when 4- to 8-cells ET was performed on the fourth or fifth day of progesterone administration in artificial cycles in donation cycles (29). In this duration, the days of progesterone supplementation were more than days of embryo.

It is suggested that one of the causes of recurrent implantation failure is displacement of implantation window (5). The aforementioned works focused on the number of progesterone administration days and not on matching the day of endometrium and embryo. It is possible that when embryos were transferred, the endometrium is not synchronized with embryo. In other words, the embryo misses the implantation window, especially in late transfer cycles. It means when embryo reaches the blastocyst stage in the uterus and is ready for implantation, the implantation window has either not started or had already finished. In this regards, Chimote and colleagues showed that the synchrony between the stage of blastocyst transferred and the endometrial preparedness is important for clinical outcomes. They determined this synchronization with respect to the day of menstrual cycle (30). We recently showed that overnight culture before the transfer of warmed embryo is not essential for FET cycles (31). Therefore, our study suggested that at the time of embryo thawing, attention should be given to the day of embryo and the number of days of progesterone administration. The major drawback of our study was the retrospective design and limited number of FET cycles. The endometrial thicknesses of all the patients were recorded as ≥ 8 mm. It is not our protocol to measure endometrium thickness on the ET day. Furthermore, it is suggested to measure the progesterone levels in serum for determining the precise synchronization between endometrium and embryo stage in FET cycles.

## 5. Conclusion

Higher rates of pregnancy and live birth were achieved with day-3 ET after three days of progesterone administration in FET cycles. Also, ET of day-4 embryo after three days of progesterone exposure had a significantly lower chemical pregnancy, clinical pregnancy, and live birth.

##  Conflict of Interest

The authors declare no conflict of interest.
